# Supplementary material for: Through the big top: An exploratory study of circus-based artistic knowledge translation in rural healthcare services, Québec, Canada
Source: PLoS One. 2024 Apr 18;19(4):e0302022. doi: 10.1371/journal.pone.0302022 (PMC11025836; doi:10.1371/journal.pone.0302022)
Supplement: Attachment — Submitted filename: Responses-to-reviewers.docx [file pone.0302022.s002.docx]

Dear members of the revision committee,

We would like to express our gratitude to the editors and reviewers for their valuable time and insightful comments on the manuscript we submitted. Below, we provide responses to each comment made by the editors and reviewers and detail the corresponding modifications made to the manuscript. We appreciate this collaborative process, which enhances the quality of our manuscript and moves us forward in our goal of submitting to PlosOne.

**Journal Requirements:**

1.Ensure that your manuscript meets PLOS ONE's style requirements. Changes were made according to guidelines: the label “Image” was changed to “Figure,” figure file names and citations were changed to fit requirements, changes were made to headings, blank lines were deleted in tables 1 and 2.

**Editors’ revision:**

1-2. Depositing protocol and data in a repository. Thank you for the information, this will be considered.

3.‘Funding Information’ and ‘Financial Disclosure’ sections. After verification, it appears the grant numbers are correct. However, in the submission form a change was made from “Fonds de Recherche du Québec-Nature et Technologies” to “Fonds de Recherche du Québec- Société et Culture” (Quebec Research Funds- Nature and Technologies to Society and Culture). The funding information was deleted from the manuscript as advised. Also, an updated financial disclosure statement was added in the cover letter in the fourth paragraph of a new version of the cover letter. In the new version of the cover letter, the opening sentence was modified to “Please accept the enclosed **REVISED** manuscript entitled “Through the Big Top: An Exploratory Study of Circus-Based Artistic Knowledge Translation in Rural Healthcare Services, Québec, Canada" for consideration for publication as an original article in Plos One.”

4. Competing interest section.  The sentence: «The authors have declared that no competing interests” was included in the last paragraph of the cover letter.

5. Data Availability Statement in the submission form. The article includes all relevant data except for the French verbatim transcripts, which can be obtained upon request. Therefore, the statement "All data are in the manuscript and/or supporting information files" was revised to the Data Availability Statement.

6. Validated ORCID iD. Completed with submission.

7. Copyrighted images for “Image” 2 and 3, now labelled “Figure” 2 and 3 in the new manuscript. The authors are the owner of the material. The following text was included in the manuscript in this format for pages 9, 12, 13, 14, 15 and 28 :“Reprinted from [ref] under a CC BY license, with permission from [name of publisher], original copyright [original copyright year].”
 
 8. Change label Images for Figures. Completed in text and figures legends for figures 1 to 5 , and figure legend only for figure 6. Pages 9, 12, 13, 14, 15, 28.

9. Consent Figure 4. Figure 4 features artists from the circus show intervention, not patients or participants in the study. The broadcasting of the show and images is stipulated in their contract. Since they are not study participants, the methods section and ethics statement remained unchanged.

10. Review reference list. The review was completed. No changes were made to the list.

**Reviewers' Comments**

Provide data as part of the manuscript or its supporting information. Please refer to previous response regarding comment #5 in the editor's comments section.

**Reviewer #1:** 

1. Quoted statement before the introduction section. Opening quotes can be a way of setting the table to what the article will be addressing, in a more poetic or philosophical approach. After reflection, we deleted the quote since it can bring confusion.

2. More detailed introduction to knowledge translation as a method. Completed, page 4. In the first paragraph of the introduction, the definition of knowledge translation is provided as follows: “In the context of Canadian healthcare, knowledge translation is defined as a dynamic and iterative process that includes synthesis, dissemination, exchange and ethically-sound application of knowledge to provide Canadians more effective healthcare services and products and strengthen the healthcare system”. To enhance clarity, the following sentence was included in the manuscript: “It aims to move important health research evidence into the hands of people and organizations who can put it into practice.”

3. Reconsider the use of "KT" as an abbreviation for "Knowledge translation".

To enhance readability, we replaced the abbreviations “KT” with “knowledge translation” and “ABKT” with “arts-based research translation” throughout the text.

4. Integrate "Objective" and "Hypothesis" sections directly into the introduction. Completed, page 6.

5. Include a flowchart in the "Procedures" subsection. To provide clarity, the legend of the figure labelled “Methodology Timeline” (Figure 5) on page 15 was updated to “Procedure Flowchart.'"

6.English expressions and academic standards. We revised the entire text to conform with academic standards and adhere to the publishing guidelines of PlosOne. This involved rectifying terminology such as "images" and "figures," as well as refining section headings and overall formatting. Our goal was to ensure that the language used was appropriate for an interdisciplinary project merging aspects of circus arts with healthcare research.

**Reviewer #2:**

Literature review of the investigation in a separate chapter. Thank you for the comment and the reference. Indeed, exploring the application possibilities of knowledge management is an interesting avenue for such projects. A literature review was recently submitted as part of first author’s PhD project. In the case of the manuscript submitted here, special intention was paid to incorporate relevant references. While the 65 references included in the manuscript may not constitute a comprehensive literature review, we believe they cover a wide range of topics related to knowledge translation and arts-based knowledge translation research.

Visual presentation of the results using figures. The comment is very relevant. We are indeed developing additional visual, creative and participatory tools to disseminate the results of this research. Regarding this manuscript, after discussion with our co-authors, we opted to present the results in a classic format using words and tables instead of visual figures. This decision aligns with our goal of establishing a foundational reference point.

We wish to thank you again for the comments, encouragement, and support.

We look forward for the next steps.

Richard Fleet
